# Modulation of 1,2-Dicarbonyl Compounds in Postprandial Responses Mediated by Food Bioactive Components and Mediterranean Diet †

**DOI:** 10.3390/antiox11081513

**Published:** 2022-08-03

**Authors:** Nadia Cruz, Marcos Flores, Inés Urquiaga, Felipe Ávila

**Affiliations:** 1 Escuela de Nutrición y Dietética, Facultad de Ciencias de la Salud, Universidad de Talca, Campus Lircay, Talca 3460000, Chile; nadia.cruz@utalca.cl; 2 Departamento de Ciencias Básicas, Facultad de Ciencias, Universidad Santo Tomás, Talca 3460000, Chile; marcosflores@santotomas.cl; 3 Center for Molecular Nutrition and Chronic Diseases, Pontificia Universidad Católica de Chile, Casilla 114-D, Santiago 8331150, Chile; iurquiaga@bio.puc.cl

**Keywords:** methylglyoxal, glyoxal, 3-deoxyglucosone, postprandial studies, advanced glycation end products, polyphenols, dietary fiber, Mediterranean diet

## Abstract

Glycoxidative stress with the consequent generation of advanced glycation end products has been implied in the etiology of numerous non-communicable chronic diseases. During the postprandial state, the levels of 1,2-dicarbonyl compounds can increase, depending on numerous factors, including characteristics of the subjects mainly related to glucose metabolism disorders and nutritional status, as well as properties related to the chemical composition of meals, including macronutrient composition and the presence of dietary bioactive molecules and macromolecules. In this review, we examine the chemical, biochemical, and physiological pathways that contribute to postprandial generation of 1,2-dicarbonyl compounds. The modulation of postprandial 1,2-dicarbonyl compounds is discussed in terms of biochemical pathways regulating the levels of these compounds, as well as the effect of phenolic compounds, dietary fiber, and dietary patterns, such as Mediterranean and Western diets.

## 1. Introduction

**Figure 1:**
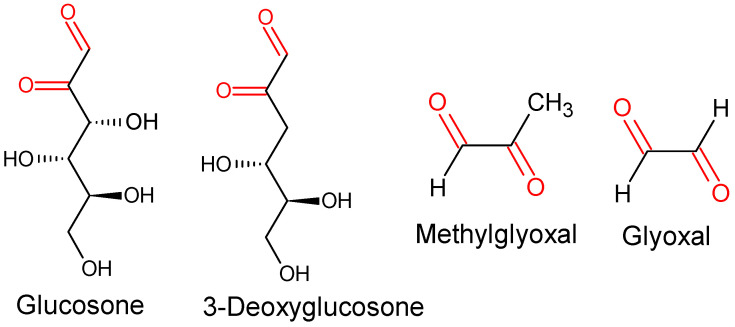
Chemical structure of some 1,2-dicarbonyl compounds of physiological relevance. The carbonyl group in each molecule is shown in red.

Electrophilic compounds are relatively unstable electron-deficient molecules that can react with nucleophiles (molecules that possess a reactive electron pair), generating covalent bonds [1]. Electrophiles can react with nucleophilic atoms of numerous molecules with physiological relevance, including amino acids, peptides, proteins, and nucleic acids, among others. Endogenous levels of electrophilic compounds can have diverse origins, including atmospheric contaminants, metabolic reactions, non-enzymatic oxidation of amino acids, lipids and carbohydrates, etc. [2,3]. Among electrophilic molecules, 1,2-dicarbonyl compounds (i.e., glyoxal, methylglyoxal, 3-deoxyglucosone, glucosone, etc.) are relevant (Figure 1) since some of them have been proposed to be associated with the etiology of numerous chronic diseases, such as type 2 diabetes and diabetic-related complications [4,5,6,7,8], rheumatoid arthritis [9], age-related cataract [10], and chronic kidney diseases [11,12], among others. α-Dicarbonyl compounds have been detected in numerous body fluids such as plasma [13], cerebrospinal fluid [14], saliva [15], and urine [16,17], among others. The analysis of serum or plasma levels of numerous 1,2-dicarbonyl compounds have been shown to be increased in patients with chronic diseases, such as type 1 and 2 diabetes and chronic kidney disease, among others, compared to healthy control subjects [4,13,18,19,20,21]. Moreover, an increase in plasma methylglyoxal levels have been associated with cardiovascular disease [22] and even mortality in type 2 diabetic subjects [4]. The plasmatic levels of some 1,2-dicarbonyl compounds, such as glyoxal, methylglyoxal, and 3-deoxygluosone (Figure 1), can increase from fasting to the postprandial state, depending on numerous factors, including the health of volunteers, age, food composition, and interaction during the digestion processes [23].

Although endogenous plasmatic levels of glycating compounds such as glucose or fructose are several orders of magnitude higher (approx. 150,000 times when fasting, for healthy subjects) than plasmatic levels of 1,2-dicarbonyl compounds, these compounds are up to 20,000-fold more reactive than glucose in glycation reactions [24]. 1,2-Dicarbonyl compounds can react readily with nucleophilic side chains of proteins or α-amino groups of amino acids, giving rise to advanced glycation end products (AGEs), which can accumulate during aging, especially in tissues containing long-lifetime proteins [25,26]. Much evidence has associated the generation of AGEs in proteins with chronic diseases such as cataract, Alzheimer’s, and cardiovascular diseases including atherosclerosis, among others [27,28,29,30,31]. One of the most important characteristics related to the etiology of these diseases is the fact that crosslinked proteins containing AGEs can be resistant to proteolytic degradation and consequently accumulated in tissues [27,32,33]. Therefore, postprandial generation of 1,2-dicarbonyl compounds can be an important pathway contributing to the endogenous accumulation of AGEs, considering that humans spend up to three quarters of each day in the postprandial state [34] and taking into account the relevance of dicarbonyl compounds in chronic disease etiology. The purpose of this review was to summarize the role of the nutritional composition of foods and, in particular, food bioactive compounds in a Mediterranean diet during the postprandial modulation of 1,2-dicarbonyl compounds. 

## 2. 1,2-Dicarbonyl Compounds in the Postprandial Period: Dietary Sources versus Metabolic Generation

α-dicarbonyl compounds can be detected in circulation, showing an increase in type 2 diabetic subjects when fasting, but they increase acutely, especially during the postprandial state. Table 1 shows that levels of methylglyoxal and 3-deoxyglucosone are increased after the intake of glucose or other carbohydrate-rich meals. It has been shown that, typically, the postprandial glycemic peak after glucose intake in healthy volunteers occurs between 15 and 30 min [35,36]. Whereas a high variability in the glycemic peak time after glucose intake has been reported for diabetic subjects, ranging from 60 to 150 min postprandial [36,37]. The lag time in the glycemic peak observed among type 2 diabetic subjects has been associated with pancreatic β-cell dysfunction [36,38,39]. A wide range of postprandial methylglyoxal peak times (from 30 to 120 min) have been reported among different studies, but also within the same studies with subjects with different impairment degrees in the glucose metabolism (Table 1). Whether a lag time in postprandial methylglyoxal peaks occur in a similar way to the lag time in postprandial glycemic peaks observed in diabetic subjects has not been systematically assessed. However, the analysis of previously published studies suggest that this fact might also occur. Maessen et al. showed that the intake of 75 g of glucose produces a shift in the postprandial glycemic peak from 30 min in healthy subjects to 60 min in type 2 diabetic subjects [40]. This fact was concomitant with the postprandial time in which the mean values of methylglyoxal reach maximum levels, corresponding to 30 and 60 min after intake, for healthy subjects and type 2 diabetics subjects, respectively [40] (Table 1). Postprandial peak time for nutrient detection on blood depends on food composition [35], food matrix [41], digestion processes, gastric emptying [42], small intestine motility [43], transport from gastrointestinal tract [44], gut microbiota activity, colonic transit, and nutritional composition of previous ingested meals [45], among other factors. Considering glucose intake, a lag time could be expected from glycemic peak levels to the methylglyoxal peak if main the blood source of methylglyoxal arises from glucose metabolism. A similar response has been shown to occur for other glycolytic products, such as pyruvate or lactate [46,47]. The postprandial metabolic changes were evaluated in 19 postmenopausal women after intake of a meal composed of refined wheat bread (50 g carbohydrates, 2.7 g total dietary fiber, 9 g protein, and 5.2 g fat), 40 g of cucumber, and 300 mL of non-caloric orange drink [46]. In this study, it was found that pyruvate and lactate peaks were detected at 45 and 60 min postprandial, whereas the glycemic peak was detected at 30 min postprandial [46]. In addition, the analysis of metabolic changes after a glucose tolerance was test carried out with 5300 individuals showed approximately 30 min of lag time from glucose to pyruvate and lactate peaks [47]. More studies are necessary to clarify the postprandial kinetic behavior of dicarbonyl compounds, which can also give insight into dicarbonyl compound metabolism. Moreover, the heterogeneity of the maximum values for glucose and methylglyoxal (Table 1) highlight the relevance to use the area under the curve to compare the postprandial effects between two or more treatments (meals).

On the other hand, Table 1 shows that mean values of the fasting levels of 3-deoxyglucosone (3-DG) are higher compared to mean values of the fasting levels of methylglyoxal. Mean values ranged from 103 to 1619 nM for 3-DG and from 79 to 392 nM for methylglyoxal (Table 1). Compared to the fasting levels, postprandial mean values of methylglyoxal increased from approximately 14 to 37%, while for 3-DG, the postprandial mean values increased from approximately 7 to 26% (Table 1). It should be noticed that almost all test meals in which postprandial levels of methylglyoxal or 3-DG were determined are mainly composed by carbohydrates (Table 1). Levels of methylglyoxal or glyoxal could also increase by the intake of polyunsaturated fatty acid, since it has been reported that these dicarbonyl compounds are also reaction products from lipid peroxidation [48]. However, in vitro analyses of edible oils heated at 200 °C for 1 h, including tuna, salmon, cod liver, soybean, corn, and olive oils, showed that malondialdehyde was the main reaction product compared to the levels of glyoxal or methylglyoxal [49]. 3-DG was not detected when a ternary mixture of Lys: Glucose: fatty acid (oleic acid, linoleic acid, or linolenic acid) were exposed to heat at 180 °C for 30 min [50]. The analysis of 1,2-dicarbonyl compounds in 173 commonly consumed foods showed that 3-DG was the most abundant dicarbonylic compound compared to the levels of methylglyoxal or 3-deoxygalactosone [51]. The absorption of 3-DG from foodstuffs has been assessed in humans by means of radiolabeled Carbon-14-3-DG. It was found that the absorption rate is very slow, and even when 3-DG was absorbed, it was quickly metabolized to 3-deoxyfructose by the enzyme 2-oxoaldehyde reductase [52]. This fact is consistent with a cross-sectional study carried out with 2566 subjects, in which the food intake of 3-DG, determined by a food frequency questionnaire, did not correlate significantly with plasmatic levels of 3-DG or skin autofluorescence [53].

**Figure 2:**
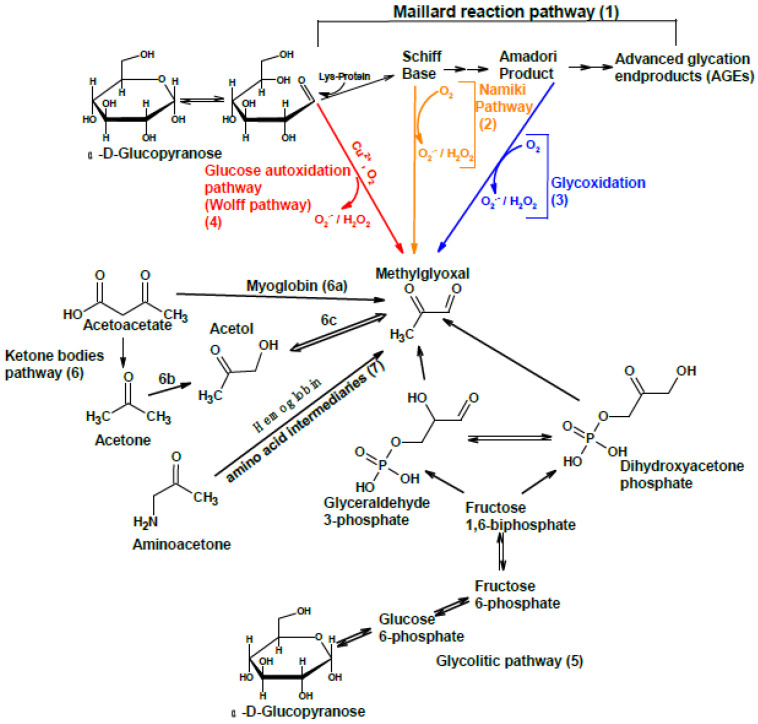
Some pathways of physiological relevance in the generation of methylglyoxal by means of enzymatic and non-enzymatic reactions.

The origin of plasmatic levels of methylglyoxal has been previously discussed [54]. Steady state concentrations of dicarbonyl compounds are determined by the rate of pathways producing it, as well as the rate of pathways that can remove these compounds. The pathways that account for endogenous methylglyoxal, other than environmental sources or those related to smoking tobacco, are mainly: (1) breakdown of early-stage Maillard reaction intermediates, such as Schiff base or Amadori products, by means of Namiki or glycoxydation pathways, respectively (Figure 2, pathways 1–3) [55,56,57,58]; (2) autoxidation of glucose and other reducing sugars (Figure 2, pathway 4) [55,59,60]; (3) lipid peroxidation of polyunsaturated fatty acids [26,48,49]; (4) UV-mediated degradation of polyunsaturated lipids [61,62,63]; and (5) oxidation of some antioxidants such as vitamin C [64,65] or β-carotene [66]. In addition to these pathways, methylglyoxal has other physiologically relevant mechanisms of generation, that include: (1) degradation of some glycolysis intermediaries, such as dihydroxyacetone phosphate and glyceraldehyde 3-phosphate (Figure 2, pathway 5) [67,68]; (2) myoglobin-catalyzed degradation of acetoacetate generated from fatty acid metabolism (Figure 2, pathway 6a) [63,69,70]; and (3) aminoacetone co-oxidation mediated by hemoglobin (Figure 2, pathway 7) [71]. In addition, the enzymatic conversion of acetone (ketone body) to acetol by means of the enzyme acetone monooxygenase or cytochrome P450 2E1 (gene CYP2E1) (Figure 2, pathway 6b) and, subsequently, to methylglyoxal by means of the enzyme acetol monooxygenase (Figure 2, step 6c) [72,73], have been described in early studies with mice and rats. 

In humans, the yield of acetone from acetoacetate has been estimated to be approx. 52% in diabetics [74] and 37% in obese humans (101.9–149.8 kg weight and 1.60–1.83 m height, N = 3) after 21 days of fasting [75]. However, the enzyme acetone monooxygenase has not been shown to be present in human tissues to date. 

On the other hand, remotion processes that can lead to methylglyoxal decrease are: (1) intracellular enzymatic detoxification throughout the glyoxalase system (composed by glyoxalase 1 and 2) [76] or aldose reductase [77]; (2) non-enzymatic reactions with proteins, lipids, and nucleic acids, as well as low molecular weight compounds, such as antioxidants, amino acids, and peptides, among others; (3) renal excretion, where an inverse correlation has been determined between plasmatic methylglyoxal levels and creatinine clearance in diabetic subjects [78]; and (4) methylglyoxal elimination by breathing, which could occur in an analogous way to acetone excretion (another C3-containing compound). However, the contribution of breath to MG elimination seems to be low, considering that methylglyoxal was not detected in the breath of non-diabetic subjects [79]. Recently, it was reported that the levels of dicarbonyl compounds detected in the breath of heart failure patients ranged from 15 to 47 pptv for glyoxal and from 4 to 23 pptv for methylglyoxal [80]. These amounts contrast with acrolein or octanal, which reported values ranging from 1350–4990 pptv for acrolein and 1230–4950 pptv for octanal [80].

In addition to the pathways described above, it has been recently shown that methylglyoxal can react non-enzymatically with acetoacetate, generating 3-hydroxyhexane-2,5-dione [81]. This metabolite was determined to have concentrations between 10–40 nM in diabetic subjects with insulin withdrawal and overnight fasting (N = 3) [81]. However, further studies are needed to determine whether this reaction can also contribute to the regulation of the postprandial levels of methylglyoxal, especially in type 2 diabetic subjects.

The effects of bioactive molecules and macromolecules present in foodstuffs, as well as dietary patterns, such as Mediterranean diets, have been evaluated on postprandial 1,2-dicarbonyl compounds. In the next section of the review, we analyze the effect of dietary fiber, phenolic compounds and Mediterranean diet on 1,2-dicarbonyl compounds. Postprandial studies, dietary interventions, animal studies, and *in vitro* experiments are discussed to give an insight on some mechanisms by which these dietary factors could exert their effects.

**Table 1:** Fasting and postprandial plasma levels of dicarbonyl compounds and methylglyoxal (MG) and 3-deoxyglucosone (3-DG) quantified in human volunteers of different characteristics.

Dicarbonyl Compound	Fasting Levels (nM) Mean ± SD	Time Max (Min)	Postprandial Levels (nM) Mean ± SD	Meal	Subjects’ Characteristics	Ref.
MG	350 ± 71	30	418.3 ^1^	75 g of glucose	Normal glucose metabolism, BMI 27.5 ± 3.9 kg/m^2^, age 58.8 ± 7.4 years, N = 279	[40]
MG	353 ± 57	60	467.3 ^1^	75 g of glucose	Impaired glucose metabolism, BMI 29.0 ± 4.3 kg/m^2^, age 59.9 ± 6.7 years; N = 120	[40]
MG	392 ± 72	60	537.9 ± 13.3 ^1^	75 g of glucose	Type 2 diabetes subjects, BMI 30.1 ± 4.5 kg/m^2^, age 60.4 ± 6.2 years; N = 92	[40]
MG	76.1 ± 3.2	30	100.8 ± 2.9	100 g of glucose	Men with prediabetes, BMI 25 and 35 kg/m^2^, age 25–50 years; N = 20	[82]
MG	157 ± 53	60 ^2^	180 ± 53	Women: 50 g carbohydrates, 13 g protein, and 13 g fat. Men: 68 g carbohydrate, 17 g protein, and 21 g fat.	Type 1 diabetics between 15 and 65 years of age, with normal renal function; N = 21	[5]
MG	293 ± 52	120	323 ± 11 ^1^	Mixed meal, energy content of 1100 kcal; 26.5 g protein (9.6%),121.0 g carbohydrates (44%) and 56.6 g fat (46.6 %)	Lean men between 18 and 65 years, nonsmoking, nondiabetic, and without cardiovascular disease, with waist circumference <94 cm; N = 25	[83]
MG	254 ± 36	30	360 ± 101 ^1^	50 g of dextrose dissolved in 300 mL of water	Men from 65 to 85 years, with exclusion criteria type 1 or 2 diabetes, stroke or heart attack in the past 6 months N = 15	[84]
MG	173 ± 34	30	201 ± 53	50 g of dextrose dissolved in 300 mL of water	Men from 18 to 35 years, with exclusion criteria type 1 or 2 diabetes, stroke or heart attack in the past 6 months N = 15	[84]
MG	297 ± 54	120	357 ± 9 ^1^	Two muffins and 300 mL low- fat milk. Meal had an energy (En) content of 1100 kcal /and provided 26.5 g protein (9.6 En%), 121.0 g carbohydrates (44 En %) and 56.6 g fat (46.6 En%)	Obese subjects, age 51.8 [45.7–60.7] years, BMI= 96.9 ± 8.4 kg/m^2^, HOMA-IR= 2.84 ± 1.38; fasting glucose = 5.64 ± 0.48 mmol/L, N = 52	[83]
MG	166 ± 23 ^1^	45, 90	192 ± 27 ^1^ (45 min); 186 ± 25 ^1^ (90 min)	75 g glucose prepared in 240 mL water.	Healthy men, 18–35 years, BMI = 19–40 kg/m^2^, fasting blood cholesterol (<200 mg/dL) and glucose (<100 mg/dL) and resting blood pressure (<140/90 mm Hg), N = 12	[23]
MG	116 ± 3	60	163 ± 3 ^1^	75 g of glucose in 473 mL of water	Prediabetic subjects (age 18–50 years), BMI 31.6 ± 0.9 kg/m^2^, fasting total cholesterol 4.6± 0.9 mM, diastolic blood pressure = 83.0 ± 1.6 mmHg, fasting glucose = 5.8 ± 0.1 mM, HOMA-IR = 2.7 ± 0.1, N = 23.	[85]
3-DG	164 ± 41	480	183 ± 45 (nmol/L)	Two croissants, 10 g butter, 40 g high-fat cheese, and 300 mL high-fat milk (3349 kJ; 50 g fat; and 56 g carbohydrates)	Normal glucose metabolism, age 60.3 ± 4.0 years, BMI 26.0 ± 3.1 kg/m^2^; N = 27	[86]
3-DG	164 ± 37	480	208 ± 40	Two slices of bread, 25 g marmalade, 30 g cooked chicken breast, 50 g ginger bread, and 300 mL drinking yogurt fortified with 45 g soluble carbohydrates (3261 kJ; 4 g fat; and 162 g carbohydrates)	Normal glucose metabolism, age 60.3 ± 4.0 years, BMI 26.0 ± 3.1 kg/m^2^; N = 27	[86]
3-DG	208 ± 60	480	236 ± 58	Two croissants, 10 g butter, 40 g high-fat cheese, and 300 mL high-fat milk (3349 kJ; 50 g fat; and 56 g carbohydrates)	Type 2 diabetes, N = 26	[86]
3-DG	210 ± 49	480	281 ± 50	Two slices of bread, 25 g marmalade, 30 g cooked chicken breast, 50 g ginger bread, and 300 mL drinking yogurt fortified with 45 g soluble carbohydrates (3261 kJ; 4 g fat; and 162 g carbohydrates)	Type 2 diabetes, N = 26	[86]
3-DG	1102 ± 156	30	1659 ^1^	75 g of glucose	Normal glucose metabolism, BMI 27.5 ± 3.9 kg/m^2^, age 58.8 ± 7.4 years; N = 279	[40]
3-DG	1191 ± 136	30–60 Almost the same	2129 ^1^	75 g of glucose	Impaired glucose metabolism, BMI 29.0 ± 4.3 kg/m^2^, age 59.9 ± 6.7; N = 120	[40]
3-DG	1619 ± 300	60	2954 ^1^	75 g of glucose	Type 2 diabetes subjects, BMI 30.1 ± 4.5 kg/m^2^, age 60.4 ± 6.2; N = 92	[40]
3-DG	103 ± 36	60	116 ± 41	Women: 50 g carbohydrates, 13 g protein, and 13 g fat. Men: 68 g carbohydrate, 17 g protein, and 21 g fat.	Type 1 diabetics between 15 and 65 years of age, with normal renal function.; N = 21	[5]
3-DG	1126 ± 228 ^1^	30	1739 ± 204 ^1^	50 g of dextrose dissolved in 300 mL of water	Men from 65 to 85 years, with exclusion criteria type 1 or 2 diabetes, stroke or heart attack in the past 6 months N = 15	[84]
3-DG	1098 ± 311 ^1^	30	1042 ± 170 ^1^	50 g of dextrose dissolved in 300 mL of water	Men from 18 to 35 years, with exclusion criteria type 1 or 2 diabetes, stroke or heart attack in the past 6 months N = 15	[84]
3-DG	993 ± 117	120	1238 ± 28	Two muffins and 300 mL low- fat milk. The mixed meal had an energy content of 1100 kcal and provided 26.5 g protein (9.6 En%), 121.0 g carbohydrates (44 En %) and 56.6 g fat (46.6 En%)	Obese subjects, age 51.8 [45.7–60.7] years, BMI= 96.9 ± 8.4 kg/m^2^, HOMA-IR= 2.84 ± 1.38; fasting glucose = 5.64 ± 0.48 mmol/L, N = 52	[83]

^1^ Approximated data, extracted from the references using webplotdigitizer. ^2^ Only determined at 60 and 120 min postprandial.

## 3. Dietary Fiber and Modulation of Glycemic Responses, 1,2-Dicarbonyl Compounds, and Advanced Glycation End Products

### 3.1. Clinical Trials Related to Dietary Fiber Supplementation

Dietary fiber intake provides many health benefits, including a decrease in the relative risk of developing coronary heart disease, stroke, hypertension, type 2 diabetes, and obesity [87]. For subjects with a high risk for developing type 2 diabetes, The American Diabetes Association recommends a minimum dietary fiber consumption of 14 g of fiber/1000 kcal per day [88] whereas, to reduce serum glucose levels, the American Dietetic Association, recommends the intake of 30–50 g of dietary fiber per day for patients diagnosed with type 2 diabetes mellitus [89]. Increased postprandial glycaemia is associated with higher levels of 1,2-dicarbonyl compounds (Table 1). In addition, type 2 diabetic subjects have higher fasting levels of 1,2-dicarbonyl compounds compared to healthy subjects (Table 1). Therefore, in this part of the review, we analyze the effect of dietary fiber intake in glycemic responses, as well as postprandial levels of 1,2-dicarbonyl compounds in human subjects.

Dietary fiber can be classified as soluble or insoluble fiber; the main sources of soluble fiber are fruits and vegetables and the main sources of insoluble fiber are cereals and whole-grain products [90]. Both types of fiber can elicit different physiologic responses that can have short-term (acute) and long-term effects, such as weight loss in overweight individuals, decrease in inflammatory responses, and effectiveness in diabetes control [90,91]. Acute effects associated with soluble fiber intake are a reduction in postprandial glucose in plasma and an increase in satiety [92]. The acute intake of some soluble fibers increases the viscosity of the fluids in the gastrointestinal tract, decreasing the rate of glucose appearance in the blood and consequently decreasing insulin secretion in diabetics [89]. Long-term effects of soluble fiber intake include a decrease in the LDL levels in hyperlipidemic subjects [93], an increase in the fermentability associated with changes in the composition of the gut microbiota in normal and overweight subjects, and weight loss in overweight and obese subjects [92]. On the other hand, the intake of insoluble fiber has also shown effects associated with reducing the risk of type 2 diabetes mellitus, an increase in satiety, the promotion weight loss, and an improvement in insulin resistance [92]. The effects of insoluble fiber intake have also been analyzed in diabetic subjects by means of supplementation of bread with wheat or oat fiber (10.2–10.6 g of fiber per portion) in a clinical trial with a crossover design carried out with 14 healthy adults [94]. In this study, the consumption of insoluble dietary fiber accelerated the acute glucose-dependent insulinotropic polypeptide (GIP) and insulin response and was further associated with a reduced postprandial glucose response on the following day upon ingestion of a control meal [94]. Although numerous clinical trials have been carried out with the aim to determine the effect of fiber on the modulations of glycemic responses [95,96,97,98,99], only few clinical studies have analyzed the postprandial effect of fiber intake on the modulation of plasmatic levels of 1,2-dicarbonyl compounds.

The effect of a dietary intervention (3 months) with brown rice was analyzed in prediabetes Chinese American subjects in a randomized clinical trial with two arms and parallel assignment [100]. The subjects that were assigned to white rice (N = 28) or brown rice (N = 30) did not show significant differences in clinical, biochemical, or anthropometric baseline characteristics, excepting for body mass index (BMI) [100]. The composition of brown rice showed a higher content of fiber (2.7 times higher than white rice) and some minerals (K and Mg) and vitamins (E and folic acid) compared to white rice [100]. The intervention with brown rice reduced the mean serum levels of methylglyoxal by 18% (P-value = 0.015) compared to baseline levels. The decrease in serum methylglyoxal levels was also significant after adjusting for weight changes [100]. In addition, the serum levels of the AGE carboxymethyl lysine (CML) were also significantly reduced (*p* = 0.026) after brown rice intervention [100]. Interestingly, no differences were observed for mean fasting glucose levels or HbA1c, but a significant decrease was observed in the levels of HOMA-IR for volunteers assigned to the brown rice arm [100]. However, these results could not only be attributed to dietary fiber increase, since the chemical composition of brown rice compared to white rice is also different for other nutrients.

A randomized clinical trial with a crossover design was carried out to determine the effect of the intake of a vegan meal (V) or a conventional meal containing meat (M) on postprandial oxidative and dicarbonyl markers, as well as other parameters, in the subjects classified as type 2 diabetes, obese with normal glucose tolerance, and control male participants (N = 20 for each group) [101]. The meals contained similar amounts of energy, carbohydrates, proteins, and total fats, but the V meal contained approximately 3.5 times more fiber and 3.9 times less saturated fatty acids content than the M meal [101]. In this study, postprandial methylglyoxal levels in plasma from the obese subjects were significantly lower after the intake of the V meal compared to the M meal [101]. Although the methylglyoxal levels were higher for the type 2 diabetics compared to the obese subjects or controls, no significant differences between meals were observed in the postprandial levels of this dicarbonyl compound for these subjects [101].

Several mechanisms by which dietary fiber can decrease endogenous levels of AGEs or 1,2-dicarbonyl compounds have been proposed. In the following part of the review, we describe the literature aimed at determining the mechanisms by which dietary fiber can modulate the number of AGEs and 1,2-dicarbonyl compounds.

### 3.2. Scavenging of 1,2-Dicarbonyl Compounds by Dietary Fiber

The effect of in vitro digestion on the scavenging efficacy of reactive carbonyl species by bound-polyphenol-rich insoluble dietary fiber (BP-IDF) in blackberry pomace, red cabbage, and wheat bran was evaluated [102]. In this study, three BP-IDF sources showed high scavenging efficacy for electrophiles such as glyoxal, methylglyoxal, acrolein, and malondialdehyde compared to alkali-hydrolyzed IDF (both hydrolyzed for 4 h and 20 h), demonstrating that polyphenols bound to insoluble dietary fiber scavenged reactive carbonyl species [102]. Additionally, Mesias et al. demonstrated in vitro a methylglyoxal trapping capacity mediated by Arabica and Robusta coffee silverskin extracts. The aqueous extracts of coffee silverskin contained soluble and insoluble fiber, melanoidins, phenolics, and other compounds [103]. These extracts consequently showed an antiglycan capacity, evaluated in vitro by means of a glycation assay carried out with BSA–glucose or BSA-MG [103]. Other components of foods related to dietary fiber, such as melanoidins [104,105], have also shown antiglycating activity [103]. In vitro gastric digestion has shown that 1 mg/mL of high-molecular weight coffee melanoidins (HMWCM) scavenged more than 40% of α-dicarbonyl compounds [106]. Similar effects were reported with cocoa melanoidins, which scavenged α-dicarbonyls such as GO and MG [107]. Consequently, these authors also showed an inhibitory effect on the formation of the AGEs, CML, and MG-H1 in a milk-model system during incubation at 35 ◦C for 21 days [107].

The scavenging capacity of dietary fiber has also been analyzed after in vitro gastric digestion. Li et al. evaluated the effect of simulated gastrointestinal digestion in bound-polyphenol-rich insoluble dietary fiber (BP-IDF), using catechin as a control [108]. The effect of gastrointestinal digestion was evaluated on the scavenging of reactive carbonyls species, such as methylglyoxal, glyoxal, acrolein, and malondialdehyde [108]. This study showed that, after in vitro gastrointestinal digestion, BP-IDF still retained considerable trapping activity for these compounds [108].

Soluble fiber, including pectin, has also shown antiglycating activity. R. Zhu et al., evaluated the in vitro inhibitory effect of pectin oligosaccharides (POSs) on the formation of AGES in an infant formula milk powder. The total AGEs were determined by means of an indirect enzyme-linked immunosorbent assay (ELISA), and carboxymethyl lysine (CML) and carboxyethyl lysine (CEL) were quantified using HPLC-ESI-ITMS/MS [109]. In this study, the POSs showed an inhibitory effect on the formation of Nε-carboxymethyllysine (CML), Nε-carboxyethyllysine (CEL), and total AGEs in the formula supplement under accelerated storage at 65 °C compared to fresh oligosaccharide-free milk and Fructo-oligosaccharide/Galacto-oligosaccharide supplementation milk powder [109].

### 3.3. Dietary Fiber, Gut Microbiota, and Advanced Glycation End Products

In vitro simulated human gastrointestinal digestion of dietary fiber extracted from spent coffee grounds of Coffee arabica L. (FSCG) and incorporated in biscuits reduced the total AGE content (determined by ELISA) by 74 and 83% for biscuits containing 3 g or 5 g per serving of FSCG, respectively, compared to a non-dietary fiber-enriched biscuit [110]. Another study was carried out with C57BL mice (4 weeks old, 16–18 g) that were assigned to four different diets, namely (1) a control diet (Diet 1), (2) a red meat with low fiber diet (Diet 2), (3) a red meat diet supplemented with dietary fiber (HF) (Diet 3), and (4) the HF diet (Diet 3) but with an AMPK-inhibition diet (Diet 4). The authors concluded that Diet 2 could promote the accumulation of methylglyoxal and glycine on cecal digesta and that diets rich in fiber (Diets 3 and 4) decreased this accumulation [111]. The authors indicated that this decrease in the accumulation of methylglyoxal could be due to a shift in gut microbiota substrate for carbohydrate metabolism from glucose to dietary fiber (mainly arabinoxylan) [111].

In an 11-week open-label clinical trial, 30 adult volunteers with normal weight consumed one daily serving of a commercially available mixture of white kidney bean extract (*Phaseolus vulgaris*), dietary fiber, ß-carotene, and noni fruit pulp (*Morinda citrifolia*) immediately before consumption of their largest meal of the day. The results showed a 10.6% decrease in AGE skin autofluorescence compared to baseline values (day 0) [112].

The effect of fiber intake on plasma AGE levels might be related to the capacity of this bioactive food component to modulate the transport of dietary AGEs from the intestinal lumen to circulation. It has been determined that the intestinal transporter PepT1 can transport some dipeptides derived from the Maillard reaction, including AGEs, such as CML and carboxyethyl lysine (CEL), from the apical membrane of Caco-2 cells to the basolateral membrane, which were detected in their hydrolyzed form (occurred intracellularly) [113,114]. The effect of butyrate, a short-chain fatty acid produced by bacterial fermentation of carbohydrates and dietary fibers, was evaluated on human intestinal epithelial Caco2-BBE cells [115]. It was found that butyrate induced hPepT1 promoter activity and mRNA and increased protein expression levels as assessed by luciferase assay, real-time RT-PCR, and Western blot, respectively [115]. Furthermore, in this study, it was observed that treatment of mice with 5 mM butyrate added to drinking water for 24 h increased colonic PepT1 mRNA and protein expression levels, concluding that butyrate increases PepT1 expression in colonic epithelial cells [115]. The effect of various dietary fibers on PePT1 was assessed in an in vivo study carried out with 100 piglets, which were assigned to four diets for 30 days. The diets included 10% of the following fiber sources: wheat bran (WB), maize fiber (MF), soybean fiber (SF), or pea fiber (PF). After the intervention period, the jejunum was removed to determine PepT1 mRNA expression [116]. It was observed that supplementation with PF increased transcription of PepT1 in mid-jejunal mucosa compared to the MF-supplemented diet, concluding that some types of fibers can increase the expression of this transporter [116]. In a similar study, 30 piglets were randomly assigned to one of five treatments for 4 weeks, namely (1) a control diet; (2) a 1% insoluble dietary fiber diet (IDF); (3) a 1% soluble dietary fiber diet (SDF); (4) a 0.75% insoluble fiber + 0.25% soluble fiber diet during the first two weeks with a 0.25% insoluble fiber + 0.75% soluble fiber diet during the last two weeks (CRMDF), or (5) a 0.5% insoluble fiber + 0.5% soluble dietary fiber diet or MDF. In this study, no significant differences in the mRNA levels of PePT1 were observed after supplementation with IDF, SDF, and CRMDF compared to the control [117]. Recently, it was reported that fish proteins glycated with glucose were included in the diet of hyperlipidemic rats—who were assigned to different diets for four weeks and compared to the control diet (fish proteins)—at a protein concentration of 6% (low-glycated proteins) and 12% (high-glycated proteins) [118]. It was found that butyrate in cecal content increased 1.59-fold for the low-glycated diet and 1.67-fold for the high-glycated diets compared to the control diet [118]. High-glycated diets reduced inflammation markers (IL-1β and IL-6) in plasma, but paradoxically, increased plasma levels of AGEs [118]. More studies are necessary to determine the mechanisms by which dietary fiber could regulate plasmatic levels of 1,2-dicarbonyl compounds and AGEs.

## 4. Phenolic Compounds and Their Effect on Dicarbonyl Compounds and Advanced Glycation End Product Formation

### 4.1. Effect of Polyphenols on Dicarbonyl Compounds: Postprandial Studies

Phenolics are naturally occurring compounds present in many foods, such fruits and vegetables, cereals, legumes, and infusions, among others [119]. Phenolic compounds can be classified as flavonoids and non-flavonoids, where the flavonoid compounds possess in their structure two aromatic rings (A and B) and one heterocycle ring (C), and non-flavonoid metabolites consist of phenolic acids, stilbenes, tannins, and lignins [120]. The consumption of these compounds has been related to the prevention of chronic diseases such diabetes, cardiovascular diseases, and cancer, among others [121,122,123].

The effect of phenolic compounds on postprandial 1,2-dicarbonyl compounds has not yet been assessed in humans. However, postprandial responses mediated by meals containing a high number of phenolic compounds on the 1, 3- dicarbonyl compound, malondialdehyde, has been evaluated [124]. Malondialdehyde is an electrophilic compound arising from the lipid peroxidation of polyunsaturated fatty acid, and it has been associated with the etiopathology of cardiovascular diseases [125,126]. We have evaluated the effect of a Chilean berry concentrate (BPC-350) on the modulation of plasma malondialdehyde postprandial in eleven healthy male volunteers using a crossover design [127]. Volunteers were randomly assigned to three experimental meals, namely 250 g of ground turkey burger (GTB) + 500 mL of water (Meal 1), 250 g of GTB + 500 mL of 5% BPC-350 (Meal 2), and 250 g of GTB cooked with 6% BPC-350 + 500 mL of 5% BPC-350 (Meal 3) [127]. This study showed that, compared to Meal 1, a significant decrease was observed in the mean value of the area under the curve of the malondialdehyde/triglycerides ratio for Meals 2 and 3. Moreover, Meal 3 also presented a statistically significant decrease in the area under the curve compared to Meal 2. This fact indicates that the presence of phenolic compounds during thermal processing reduces the postprandial malondialdehyde and triglyceride levels [127]. This effect was also reported in a study by Gorelik et al., which consisted of giving two types of meals to fourteen healthy volunteers: (1) meat cutlets (MC) and (2) meat cutlets with red wine (MCRW) [128]. They showed that postprandial plasma MDA levels after the MC meal increased by 106 nmol/mL and only by 57 nmol/mL after the MCRW meal [128]. Postprandial MDA can also be increased by the intake of carbohydrate-rich meals, as shown in a study carried out with 11 overweight men who consumed three test meals: (1) high carbohydrate (HC) (Meal 1), (2) HC meal plus 0.5 g of MC (*Mesona chinensis*) extract (Meal 2), and (3) HC meal plus 1 g of MC extract (Meal 3) [129]. The extract was characterized in terms of its antioxidant activity and total polyphenol (TP) content showing ORAC values of 25.98 ± 1.30 mg trolox equivalents/mg of dried extract and 212.37 ± 5.64 mg gallic acid equivalents/g of dried extract for TP [129]. In this study, plasma MDA postprandial was estimated and it was shown that when comparing Meal 1 to Meal 3, a significant decrease in the incremental postprandial AUC of the plasma MDA level was observed [129].

### 4.2. Dietary Interventions with Phenolic Compounds and Their Effect on 1,2-Dicarbonyl Compound Levels and Advanced Glycation End Products

#### 4.2.1. Clinical Trials

The long-term effect of dietary supplementation with phenolic compounds has been assessed in humans. Xue et al. assessed the effect of dietary supplementation with 90 mg trans-resveratrol + 120 mg hesperetin per day for a period of 8 weeks in a randomized, placebo-controlled crossover study, carried out with 29 overweight and obese subjects [130]. This study showed a significant decrease in plasma methylglyoxal levels after the intervention with trans-resveratrol and hesperetin, as well as an increase of 22% in the activity of glyoxalase 1 (Glo1) from peripheral blood mononuclear cells (PBMCs) compared to the placebo [130]. The decrease in plasma MG levels was also reported in a randomized crossover study carried out with 37 healthy adults, which were assigned to a dietary supplementation with quercetin 3-glucoside (160 mg) for 4 weeks. It was shown that the treatment with quercetin 3-glucoside decreased the levels of MG by 11% compared to baseline values [131].

In a pilot study, seventy-four healthy subjects were randomly assigned to an intervention group (N = 39), consuming 600 mg per day of *Vaccinium Myrtillus* extract for 3 months, and were compared to a control group (placebo). In this study, plasma CML levels significantly decreased in the intervention group compared to the control group [132]. In a parallel group study, 100 mg per day for 12 weeks of mixed herb extract (MHE) containing *Chamaemelum nobile* (roman chamomile), *Crataegus laevigata* (hawthorne berries), *Houttuynia cordata* (dokudami), and *Vitis vinifera* (grape leaves) was orally administered to 24 adult Japanese women with high amounts of skin autofluorescence [133]. In this study, a significant reduction in plasma 3-DG was determined in the MHE group compared to basal 3-DG levels [133].

#### 4.2.2. Studies with Murine Models and In Vitro Studies Evaluating 1,2-Dicarbonyl Compounds Interaction with Phenolics

The inhibitory effect of phenolic compounds on the formation of AGEs could be partially explained because these compounds can scavenge reactive 1,2-dicarbonyl compounds, such as methylglyoxal and glyoxal [134]. Zhao et al. have analyzed the effect of the incorporation of quercetin and methylglyoxal on CD-1 mice diet during 1 week. The mice were assigned to different intervention groups: AIN-93g diet with normal drinking water (control), AIN-93g diet with 0.12% MG in drinking water (MG group), and 0.2% quercetin in AIN-93g diet and 0.12% MG in drinking water (Q-MG group). The analyses demonstrated that after 1 week, the Q-MG group showed lower levels of MG concentration in plasma and kidney compared to the MG group [135]. A decrease in MG levels was also found in plasma, kidney, and liver after a 6-week dietary intervention with different doses of dietary quercetin in CD-1 mice [135].

In another study, the concentration of rutin-MG adducts was determined in the plasma of rats after rutin was orally administered (100 mg/kg of bodyweight). Three rutin-MG adducts were quantified, showing that the concentration of 6-(1,2-propanedione)-8-(1-acetol)-rutin (adduct A) was consistently the highest in the plasma, followed by 6-(1-acetol)-8-(1,2-propanedione)-rutin (adduct B) and 6-(1,2-propanedione)-8-(1,2-propanedione)-rutin (adduct C). These results showed that adduct A was the major endogenous product detected after rutin and MG intake [136]. Additionally, in this study, the cytotoxicity in different cell lines of this adduct was evaluated. The results showed that the formation of rutin-MG adducts remarkably reduced the toxicity of MG [136]. The formation of adducts between phenolic compounds and MG was also demonstrated by Zhang et al. by means of an assessment of the oral administration of taxifolin and MG in mice. It was shown that MG was trapped by taxifolin and its metabolites in mice urine and fecal samples, in a dose-dependent manner [137]. The analysis of an in vitro simulated digestion of a snack food (containing glucose, fructose, and sucrose) showed that digestion especially increased levels in glyoxal, which decreased when incubating with three different beverages (green tea, bergamot-flavored black tea, and olive-leaf tea) [138].

It has been proposed that one of the mechanisms by which phenolic compounds could potentially help to prevent some chronic diseases is their ability to reduce AGE formation [134]. The antiglycating effect of phenolic compounds obtained from different vegetal species has been demonstrated by means of in vitro studies, using different glycating agents, including glucose and 1,2-dicarbonyl compounds [139, 140, 141]. In addition, a cookie model fortified with five selected dietary polyphenols (naringenin, quercetin, epicatechin, chlorogenic acid, and rosmarinic acid) showed inhibition against the formation of glyoxal and total fluorescent AGEs, especially those fortified with quercetin [142]. Additionally, streptozocin-induced type 1 diabetes in mice was used to study the formation of AGEs in vivo [143]. Tea polyphenols were given to mice daily, at a dose of 200 mg/kg of bodyweight for 8 weeks, and showed that the formation of AGEs decreased in a dose-dependent manner with increasing doses of tea polyphenols [143].

Maietta et al. evaluated 1,2-dicarbonyl trapping activities of Cretan tea (*Origanum dictamnus* L.) in vitro, a beverage rich in polyphenols and phenolic acids, characterized by means of RP-HPLC-DAD [144]. The results showed that the trapping capacity of dittany infusion on MG was higher than that of glyoxal in the first 24 h, and that for longer time periods (70 to 170 h), no differences between MG or glyoxal were observed [144]. Similar effects were observed in the study by Zhu et al., where MG trapping was evaluated by means kinetic analysis using trans-resveratrol, apigenin, and kaempferol or fisetin, as well as some amino acid residues [145]. This study showed that MG scavenging increased with polyphenol concentration and continued for 30 days [145]. The formation of adducts MG-polyphenols has also been shown to occur in in vivo studies [135,136,137]. On the other hand, the decrease in dicarbonyl compounds is also related to cellular responses mediated by phenolic compounds, including the activation of Glo1. We have shown that when human gastric epithelial cells were pre-incubated for 16 h with polyphenolic-enriched extracts from Chilean native berries, increased levels of Glo1 activity, concomitant with a reduction of intracellular CML levels, were observed [146]. Co-incubation (30 min) of AGS cells with polyphenolic-enriched extracts from Chilean native berries did not show an increase in Glo1 activity [146]. Different cells have shown several lag times, usually over hours, to induce an increase in the activity and/or expression levels of enzymes regulated by nuclear factor erythroid-derived 2-like 2 (Nrf2) [147,148]. Higher levels in the activity of the Nrf2-regulated enzyme, Glo1, have been shown to decrease the intra- and extracellular levels of MG in different cells [149,150]. However, the postprandial action of phenolic compounds to reduce the levels of methylglyoxal or glyoxal by means of an increase in the expression levels and activity of Glo1 might be reflected hours after the intake and not minutes postprandial.

## 5. Mediterranean Diet and Postprandial Effects Associated with the Levels of Dicarbonyl Species

### 5.1. Mediterranean Diet as a Healthy Dietary Pattern 

The Mediterranean diet (MedDiet) has been assessed and validated internationally as one of the healthiest dietary patterns [151,152,153,154]. Conceptually, the term MedDiet originated from the classical study of the Seven Countries Studies conducted by Ancel Keys. In this study, food intake was assessed to determine its association with coronary heart disease in cohorts from Greece, Italy, and former Yugoslavia, and compared to non-Mediterranean cohorts in Northern Europe, Japan, and the USA [155]. Numerous studies indicate a beneficial effect of the MedDiet on human health [151,152]. Cross-sectional and prospective cohort studies including several meta-analyses have related the MedDiet to a lower prevalence and incidence of numerous non-communicable chronic diseases and related conditions (e.g., dyslipidemias, obesity, metabolic syndrome, type 2 diabetes, cardiovascular diseases, cancer, and neurodegeneration). In addition, beneficial effects related to the MedDiet, including a reduced overall mortality, increased longevity, and improvement in quality of life, have also been reported [156,157,158]. Clinical intervention trials for primary and secondary prevention, in which the diet was modified by increasing their adherence to the MedDiet, have been shown to decrease the incidence of numerous chronic diseases [155,159].

The MedDiet is characterized by the consumption of foodstuffs including a (1) high intake of vegetables, fruits, whole grains, legumes, nuts, herbs, and spices, olive oil, white meats, fish, and seafood; (2) moderate intake of red wine and fermented dairy products; and (3) low consumption of butter, whole milk, sugar, and red meats [160]. 

Oxidative stress and chronic inflammation constitute primary risk factors related to the aging process and chronic non-communicable diseases [161,162]. Hence, the mechanisms associated with the healthy effects of the MedDiet have been shown to be related to a reduction of markers of inflammation and oxidative stress, as well as melioration in blood lipid profile, insulin sensitivity and endothelial function, and antithrombotic effects [155,163,164,165]. These effects have been attributed to bioactive ingredients such as fiber, polyphenols, and mono- and poly-unsaturated fatty acids (mainly oleic acid from olive oil) [165,166]. In this part of the review, we discuss the literature related to the effects of the MedDiet and some of its characteristic foodstuffs on the postprandial and fasting 1,2-dicarbonyl compounds and related electrophiles.

### 5.2. Postprandial Effects of Mediterranean and Western Diets in the Generation of 1,2-Dicarbonyl Compounds and Related Compounds

Olive oil (*Olea europaea*) is one of the most emblematic foodstuffs present in the Mediterranean diet, which possesses a high amount of monounsaturated fatty acids, mainly oleic acid (approx. 80%) [167]. In people with metabolic syndrome, it was reported that a diet high in monounsaturated fatty acids (MUFAs) limits postprandial oxidative stress more than diets that are low in fat and high in complex carbohydrates and saturated fatty acids (SFAs) [168,169]. In line with this, the MedDiet, which is rich in monounsaturated fatty acids (MUFA) and minimally processed natural foods, was found to reduce postprandial oxidative stress and inflammation [170]. However, the Western lifestyle induces a chronic state of systemic low grade-inflammation, which is involved in the pathogenesis of a wide range of chronic diseases and related conditions such as obesity, metabolic syndrome, type 2 diabetes, non-alcoholic fatty liver disease (NAFLD) and atherosclerosis [170]. It was reported that intake of virgin olive oil by obese subjects induces a lower postprandial mRNA expression in inflammation markers (MIF and JNK1) in PBMCs compared to sunflower oil, even after thermal treatment (180 °C, 5 min for 20 heating cycles). This effect could be attributed to different factors as the authors indicated, including the different composition of fatty acids and polyphenol content [168], but also to the generation of different amounts of electrophiles because of the thermal processing. The generation of 1,2-dicarbonyl compounds (glyoxal and methylglyoxal) and malondialdehyde, as a consequence of the thermal degradation of olive oil, was analyzed and compared to other oils rich in ω-6 fatty acids, which are typically consumed in Western diets [171]. It was found that thermal treatment (200 °C, 1 h or 60 °C for 3 or 7 days) of olive oil generates lower amounts of malondialdehyde, glyoxal, and methylglyoxal compared to oils with a high amount of polyunsaturated fatty acids, including salmon, tuna, and cod liver oils [49]. In vitro gastric digestion experiments were carried out by J. Kanner and colleagues, in which the electrophilic compound, malondialdehyde, was quantified after turkey meat digestion in the presence of olive oil and fish oils [171]. It was found that olive oil decreased the mean levels of malondialdehyde by approximately 60% (from 121.7 ± 3.1 to 48.2 ± 1.3 μM), which was attributed to oleic acid rather than polyphenol content [171]. In contrast, fish oils increased the levels of MDA by approximately five times (from 96.2 ± 3.6 to 514.2 ± 6.7 μM) [169]. The postprandial modulation of the levels of 1,2-dicarbonyl compounds induced by olive oil and the modulation by phenolic compounds deserves further study, especially considering the effect of thermal treatment, which has also been pointed out as an important factor in the effects induced by the MedDiet [172] and Western diets [173]. 

Some studies support the contribution of 1,2-dicarbonyl compounds, particularly methylglyoxal (MG) and AGEs such as N-carboxymethyllysine (CML), in the origin and progression of chronic diseases and during the aging process, increasing oxidative stress and inflammation [174,175]. Although endogenous AGE formation represents a minor component of the total body pool of AGEs, dietary AGEs (dAGEs) are one of the most important sources of AGEs [176]. The evidence suggests that an AGE-restricted diet is an effective way to reduce the body’s total AGE concentration and decrease oxidative stress and inflammation in different populations [177].

Lopez-Moreno et al. 2016, reported a crossover study in which 20 participants (aged ≥65;) were randomly assigned to receive three isocaloric diets for periods of 4 weeks for each diet: a Mediterranean diet (24% monounsaturated fatty acids, MUFA; provided by virgin olive oil); a Western diet rich in saturated fat (22% saturated fatty acids, SFA); and a low-fat, high-carbohydrate (omega-3) diet enriched in omega-3 polyunsaturated fatty acids (PUFAs) of vegetable origin (8% PUFA with 2% alpha-linolenic acid). The postprandial challenge was a breakfast with the same fat composition than the previous diet period. It was shown that consumption of a MedDiet reduces AGEs and increases antioxidant defenses in the fasting and postprandial states, evidenced by lower serum N-carboxymethyllysine (sCML) and methylglyoxal (sMG) levels, higher levels of the AGE receptor-1 (AGER1) and Glo1, and lower mRNA levels of the receptor for AGEs (RAGE) compared to a Western-type diet rich in SFA with an intermediate effect of an omega-3 diet [178].

These authors (Lopez-Moreno et al. 2018) continued studying whether supplementation of the MedDiet with coenzyme Q10 (CoQ) was of further benefit. They reported that the beneficial effect of the MedDiet in the postprandial phase was accentuated by adding CoQ. The MedDiet + CoQ produced lower postprandial sMG and sCML levels compared to the solitary MedDiet, and lower postprandial levels of sAGEs after the MedDiet compared to a Western-type diet [169]. Interestingly, sMG levels decreased significantly during the postprandial phase compared to the fasting state in the MedDiet + CoQ group but were unchanged in the MedDiet group and were even increased significantly in the Western-type diet group [169]. Furthermore, in PBMCs, the MedDiet and Med + CoQ produced higher fasting and postprandial AGER1 mRNA levels and lower fasting and postprandial RAGE mRNA levels compared to the Western-type diet [169]. However, significantly higher postprandial AGER1 mRNA levels after the MedDiet + CoQ compared to the MedDiet was observed [169]. No differences were found on fasting and postprandial RAGE mRNA levels between the MedDiet + CoQ and MedDiet. Conversely, the MedDiet and MedDiet + CoQ produced a significant increase in AGER1 mRNA levels during the postprandial state compared to the fasting state, whereas consumption of the Western-type diet resulted in a significant decrease of postprandial AGER1 mRNA levels in comparison with the fasting state [169].

Additionally, in PBMCs, the MedDiet and MedDiet + CoQ produced higher fasting and postprandial Glo1 mRNA levels compared to the Western-type diet. Conversely, the MedDiet + CoQ produced higher postprandial Glo1 mRNA levels compared to the Med Diet. In addition, both the MedDiet and MedDiet + CoQ produced a significant increase in Glo1 mRNA levels during the postprandial phase compared to the fasting state but not after the Western-type diet [169].

Univariate analysis showed a significant positive correlation between AGER1 mRNA levels and nuclear factor erythroid-derived 2-like 2 (Nrf2) mRNA levels, nitric oxide levels and thioredoxin (Trx) mRNA levels, and a negative correlation with protein carbonyl levels and with 8-isoprostanes levels. Additionally, RAGE mRNA levels showed a positive correlation with IL-8 mRNA levels, 8-isoprostanes levels, and sMG content [169,179,180].

Consistently, during the Western-type diet, participants consumed more dAGEs than other diets, with the MedDiet providing the lowest number of food-related AGEs, and the omega-3 diet having an intermediate content level. There were no differences in the number of food-related AGEs between the MedDiet and MedDiet + CoQ consumption of dAGES. However, the supplementation with CoQ resulted in a greater decrease in endogenous AGE levels, sMG, and sCML during the postprandial state, which is likely a consequence of an increase in the gene expression related to AGE metabolism, AGER1, and Glo1 [169].

Several studies have shown that a low-AGE diet reduced circulating AGE levels and chronic inflammation or oxidative stress in patients with age-related diseases [175,181]. So, the MedDiet may have a protective effect against oxidative stress and inflammation, providing low-AGE content and reducing circulating AGEs. In addition, the MedDiet may have favorable effects because of its impact on the host defense system to restrict AGE toxicity. The circulating RAGE receptor binds AGEs and reduces extracellular AGEs, but induces oxidative stress and inflammation, thus it can be considered to be a deleterious response [182]. The MedDiet reduces fasting and postprandial RAGE mRNA levels contrary to what an SFA diet achieves. On the other hand, the MedDiet intake increases AGER1 and Glo1 mRNA levels. AGER1 is considered to be the main host defense against glycoxidants and is associated with Nrf2, which promotes the expression of antioxidant defense system.

Inhibition of AGE formation may limit oxidative and inflammatory damage in tissues, retarding the progression of chronic diseases and improving quality of life during aging [183]. The dietary pattern of the MedDiet provides a healthy nutrient intake balance: high content of antioxidants, fiber, and monounsaturated fat, adequate omega-6/omega-3 fatty acid ratio, and low refined carbohydrate, saturated fat, and terrestrial animal protein consumption [184,185]. These components could explain the antioxidant and anti-inflammatory effects of the consumption of a MedDiet.

Regarding protein consumption, the MedDiet involves a low intake of red meats (including processed red meats); these kinds of foodstuffs are recognized as pro-oxidants. Therefore, compared to Western diets, in which the consumption of these kinds of meats is frequent, the MedDiet could contribute to modulating the systemic redox status to become a less oxidative one [186]. Different approaches have been employed for the in vitro assessment of the effect of proteins from meats in the levels of 1,2-dicarboinyl compounds or the electrophilic compound malondialdehyde, including (1) the generation of 1,2-dicarbonyl compounds or their related AGEs generated as a result of thermal treatment or in vitro digestion and (2) 1,2-dicarbonyl compounds’ scavenging capacity mediated by proteins as a result of the reaction with nucleophilic centers (mainly sulfhydryl and amino groups). Steppeler et al. evaluated the effect of in vitro digestion on the malondialdehyde levels induced by different cooked meats (pork, chicken, and salmon), which contained similar amounts of fats. They showed that the amount of polyunsaturated fatty acid and not total iron was the main factor associated with the generation of malondialdehyde [187]. In this study, it was also shown that the highest amount of malondialdehyde was generated by the incubation of mince beef with fish oil [187]. On the other hand, it was also shown by other researchers that MDA was significantly and positively correlated (*p* < 0.0004) with the heme content of meats (beef, pork, and chicken) after gastrointestinal digestion (including colonic digestion) [188]. It was suggested that the iron present in red meat was associated with the increase increasing lipid peroxidation. We have recently reported that when heated alone (60 °C, 2 h), sarcoplasmic proteins from beef were the only species in which irreversible protein crosslinking was significantly increased (contrasted with controls) compared to salmon, turkey, or chicken [141]. On the other hand, Dogan et al. evaluated the capacity of methylglyoxal scavenging elicited by different foods under gastric conditions, specifically typical animal protein in the MedDiet such eggs and chicken, which caused a reduced formation of methylglyoxal by 63.3 and 90.1 %, respectively [189]. Contrastively, beef meat was the most effective to generate CML when fried (outer layer), compared to pork, chicken, salmon, and tilapia (204 °C, 20 min) [190]. These studies support the epidemiological studies in which red meat is associated with oxidative stress and chronic disease markers [191].

Oxidized proteins and amino acids are potential promoters of luminal and postprandial oxidative stress [192]. The MedDiet includes a high consumption of vegetables and fruits, virgin olive oil, and red wine in moderation, all rich in antioxidants such as polyphenols, which are dietary components implied in the protection against oxidative stress and inflammation [179]. In addition, the way of cooking in the MedDiet, using virgin olive oil to sauté lots of vegetables, such as onions, garlic, parsley, mint, oregano, and tomatoes, are an “antioxidant cocktail”. This basic recipe incorporating meat, fish, or beans could control the oxidative damage of food ingredients [193]. Additionally, vegetables and virgin olive oil together with a cup of red wine reduced the postprandial oxidative stress.

A recent narrative review focused on the phenols in red wine and virgin olive oil, discussing the evidence of their effects on oxidative stress and inflammatory markers [165].

Some studies have suggested that the adequate omega-6/omega-3 fatty acid ratio is 1–2:1, but nowadays it could be even higher than 20:1. It was observed that the high intake of omega-6 (n-6) polyunsaturated fatty acids (PUFA) in Western diets contributes to increased metabolic inflammation, as n-6 PUFA results in a higher production of eicosanoids such as leukotrienes, prostaglandins, and lipoxins compared to n-3 PUFA [194].

## 6. Conclusions

The assessment of postprandial responses of 1,2-dicarbonyl compounds is relevant because of their association with the etiopathogenesis of some chronic diseases, which has been mainly assessed for diabetes. The postprandial responses in which methylglyoxal and 3-deoxyglucosone are detected depends on many variables, including physiological state, amount and composition of food, among other parameters. This fact emphasizes the need for a systematic characterization of postprandial responses, considering the type of nutrient to be analyzed, age, and health status, among others. Postprandial parameters such as the peak time of 1,2-dicarbonyl compounds in subjects with different health statuses (after the intake of food with standardized composition) should be further assessed. This could give insights into the generation mechanisms of plasma 1,2-dicarbonyl compounds. The effect of food components such as dietary fiber or phenolic compounds indicate their potential for decreasing postprandial levels of electrophiles, but more studies are needed in particular to elucidate their effects on the levels of 1,2-dicarboyl compounds.
